# Cold adaptation regulated by cryptic prophage excision in *Shewanella oneidensis*

**DOI:** 10.1038/ismej.2016.85

**Published:** 2016-08-02

**Authors:** Zhenshun Zeng, Xiaoxiao Liu, Jianyun Yao, Yunxue Guo, Baiyuan Li, Yangmei Li, Nianzhi Jiao, Xiaoxue Wang

**Affiliations:** 1 Key Laboratory of Tropical Marine Bio-resources and Ecology, Guangdong Key Laboratory of Marine Materia Medica, RNAM Center for Marine Microbiology, South China Sea Institute of Oceanology, Chinese Academy of Sciences, Guangzhou, China; 2 University of Chinese Academy of Sciences, Beijing, China; 3 State Key Laboratory of Marine Environmental Sciences, Xiamen University, Xiamen, China; 4 Institute of Marine Microbes and Ecospheres, Xiamen University, Xiamen, China

## Abstract

Among the environmental stresses experienced by bacteria, temperature shifts are one of the most important. In this study, we discovered a novel cold adaptation mechanism in *Shewanella oneidensis* that occurs at the DNA level and is regulated by cryptic prophage excision. Previous studies on bacterial cold tolerance mainly focus on the structural change of cell membrane and changes at the RNA and protein levels. Whether or not genomic change can also contribute to this process has not been explored. Here we employed a whole-genome deep-sequencing method to probe the changes at DNA level in a model psychrotrophic bacteria strain. We found that temperature downshift induced a 10 000-fold increase of the excision of a novel P4-like cryptic prophage. Importantly, although prophage excision only occurred in a relatively small population of bacteria, it was able to facilitate biofilm formation and promote the survival of the entire population. This prophage excision affected cell physiology by disrupting a critical gene encoding transfer-messenger RNA (tmRNA). In addition, we found that the histone-like nucleoid-structuring protein (H-NS) could silence prophage excision via binding to the promoter of the putative excisionase gene at warm temperatures. H-NS level was reduced at cold temperatures, leading to de-repression of prophage excision. Collectively, our results reveal that cryptic prophage excision acts as a regulatory switch to enable the survival of the host at low temperature by controlling the activity of tmRNA and biofilm formation.

## Introduction

Temperature is a major determining factor for the selection and distribution of microbes in different habitats and these organisms may experience temperature fluctuations during seasonal changes or contact with different hosts. Microbes can be classified into hyperthermophiles/thermophiles, mesophiles or psychrotrophs/psychrophiles according to the range of temperatures in which they can grow (Hebraud and Potier, 1999). The latter, psychrotrophs and psychrophiles, are of particular importance, because they are often regarded as the most successful colonizers of the Earth's biosphere, more than three-quarters of which is occupied by cold ecosystems including ocean depths and polar regions (Feller and Gerday, 2003). Psychrotrophs (also referred to as facultative psychrophiles) are considered more versatile than psychrophiles (also referred to as obligate psychrophiles), owing to their ability to grow not only at freezing temperatures but also at the moderate temperatures occupied by mesophiles (Ray *et al.*, 1998). However, cold-shock responses are relatively better documented in mesophilic bacteria, which survive under adverse conditions through physiological responses to temperature downshifts. Adaptive processes during temperature shifts in different bacteria include changes in cell membrane properties (membrane fluidity and permeability) (Barria *et al.*, 2013), proteins (transcriptional regulators, kinases and chaperones) (Bae *et al.*, 2000; Braun *et al.*, 2007), and RNA and DNA secondary structure (Lopez-Garcia and Forterre, 2000; Phadtare, 2011).

Global stress responses, including temperature shifts, can also result in genetic variability. In particular, growing evidence suggests that movement of mobile genetic elements, activation of error-prone DNA polymerases and downregulation of DNA repair enzymes are common stress-induced strategies for mutagenesis in bacteria (Foster, 2007). Unlike eukaryotes, which primarily evolve through the modification of existing DNA, bacteria have obtained a significant proportion of their DNA via acquisition of sequences from distantly related organisms (Ochman *et al.*, 2000). Indeed, horizontal acquisition of DNA dramatically increases genetic diversity, permitting successful colonization of several niches (Biemont and Vieira, 2006). In addition, prophage-encoded virulence factors, including cholera toxin in *Vibrio cholerae* (Waldor and Mekalanos, 1996) and the Shiga-like toxin in *Escherichia coli* O157:H7 (Wagner *et al.*, 2002), contribute to pathogenesis. For instance, prophage Gifsy-2 confers a competitive advantage to its *Salmonella* host by killing competitors (Bossi *et al.*, 2003) and Lambda, Mu, P1 and P2 prophages increase *E. coli* growth under energy-limiting conditions (Edlin *et al.*, 1975, 1977; Chen *et al.*, 2005). In these cases, a lysogenic symbiotic relationship between the phage and host is created and fixation of prophage genes appears to be restricted to functions co-opted by the host (Canchaya *et al.*, 2004). Crucial to prophage gene expression are excision from and integration into the genome, steps that are mediated by recombinases through site-specific recombination between the attachment site in the bacterial genome and that in the phage genome (Huber and Waldor, 2002; Feiner *et al.*, 2015). However, prophages can disrupt the gene structure of the host on integration or excision, and when integrated into critical genes they can also serve as switches that regulate host physiology via genome excision or integration, as recently reviewed by Feiner *et al.* (2015). For example, phagosomal escape is a crucial step for *Listeria monocytogenes* infection and excision of prophage A118 from the *L. monocytogenes* genome is specifically induced during intracellular growth within phagosomes, which restores a competence gene required for efficient escape (Rabinovich *et al.*, 2012). In addition, prophage CP4-57 excision activates the motility operon during *E. coli* biofilm formation (Wang *et al.*, 2009) and prophage Pf4 excision during *Pseudomonas aeruginosa* biofilm formation leads to formation of small-colony variants with increased virulence (Webb *et al.*, 2004).

Strains of *Shewanella* have been isolated from diverse geographic habitats that are either periodically or permanently cold, including water columns and sediments of different depths (Hau and Gralnick 2007). Comparative genomic analysis of different *Shewanella* strains shows that mobile elements such as prophages, genomic islands and insertion elements comprise the predominant differences in gene content among genomes of the same species (Konstantinidis *et al.*, 2009). *S. oneidensis* MR-1 is the first *Shewanella* spp. genome to be sequenced (Heidelberg *et al.*, 2002) and this strain serves as the model organism for studying the functional repertoire of the *Shewanella* genus. *S. oneidensis* was originally isolated from sediments of Lake Oneida, New York (Myers and Nealson, 1988), a shallow freshwater system that freezes during winter, although the water temperature reaches 25 °C in midsummer (Mills and Holeck, 2001). Following a shift from an optimal growth temperature of 30 °C to near-freezing, the growth of *S. oneidensis* immediately pauses and then resumes after a lag period, which indicates that the organism is psychrotrophic (Gao *et al.*, 2006). Furthermore, *S. oneidensis* cells exhibit changes in morphology, growth rate, ultrastructure and lipid composition after a shift from 22 °C to 3 °C (Abboud *et al.*, 2005). At the transcriptional level, *S. oneidensis* responses to a temperature downshift from 30 °C to 8 °C are characterized by upregulation of genes encoding membrane proteins, DNA metabolism factors, translation apparatus components and regulatory proteins (Gao *et al.*, 2006). However, there is little information to date regarding whether genetic variability is involved in the cold adaptation of psychrotrophs.

In this study, a genome deep-sequencing approach was employed to probe genetic changes in *S. oneidensis* during temperature downshifts. Here we report the identification of a new cryptic prophage in *S. oneidensis*, named CP4So, harbored at the 3′-end of a gene encoding a transfer-messenger RNA (tmRNA). Excision induction due to a temperature downshift resulted in a mutation in the tmRNA gene that completely abolished its function. At a warm temperature, the histone-like nucleoid-structuring protein (H-NS) silenced CP4So excision by binding to the excisionase gene *alpA*, to maintain the function of the tmRNA. In contrast, CP4So excision was de-repressed at cold temperatures due to reduced levels of H-NS. The resulting prophage-excised subpopulations survived longer at cold temperatures and formed more strongly attached biofilms, and the presence of a small fraction of prophage-excised cells promoted early biofilm formation in the entire population.

## Materials and methods

### Whole-genome deep-sequencing of *S. oneidensis* cells at cold temperatures

*S. oneidensis* cells were harvested after planktonic growth for 7 days at 4 °C and total DNA was extracted using a TIANamp Bacteria DNA Kit (Tiangen, Beijing, China). Whole-genome sequencing and mutation analysis were performed using Illumina Hiseq 2500 sequencing technology and the Burrows–Wheeler Alignment tool by Shanghai Majorbio Bio-Pharm Technology Co., Ltd. Planktonic *S. oneidensis* cells grown for 1 day at 30 °C were also collected and sequenced as a control. The quality control for the whole-genome sequencing and genome alignment data are listed in Supplementary Tables S2 and S3.

### Prediction of SsrA secondary structure

The secondary structure of the wild-type SsrA and the variant SsrA was predicted using ARAGORN (Laslett and Canback, 2004) (http://mbio-serv2.mbioekol.lu.se/ARAGORN/).

### Prediction of the prophage element inserted in *ssrA*

We searched for potential phage attachment sites using the sequence from the start of the *ssrA* gene to the start of the *intA* gene and found a match of 26 nt near *SO_1440*, which is ~36 kb downstream of *ssrA*. Through a comparative genomic analysis with closely related species, including *Shewanella* sp. MR-4, *Shewanella* sp. MR-7 and *Shewanella* sp. ANA-3, the gene region from *SO_1440* to *SO_1471* was identified as a genomic island or prophage that was probably acquired through horizontal gene transfer (Supplementary Figure S9). Deletion of a U at the 3′-end of SsrA on prophage excision is predicted through simulation of prophage excision from the *S. oneidensis* host genome via a site-specific recombination as previously described (Wang *et al.*, 2009).

### Screening of the ΔCP4So strain at cold temperatures

*S. oneidensis* cells that underwent CP4So excision were obtained by PCR-based screening using cells growing planktonically at 4 °C for 7 days. Owing to the size of the prophage (36 kb), PCR using the primer pair SmpB-f and SO1439-r can generate an 869-bp fragment only after CP4So has excised from the host genome (Supplementary Table S4 and Supplementary Figure S1a and b). The excised CP4So prophage was found to form a circle, which was confirmed by a PCR-based assay followed by sequencing (Supplementary Figure S1c), but it was gradually lost, similar to the P4-like prophage in *E. coli* (Wang *et al.*, 2009). The complete lack of CP4So genes in ΔCP4So was verified by DNA sequencing using the SmpB-f and SO1439-r primers and confirmed by the loss of the genes *intA* and *SO_1444* within CP4So via PCR (Supplementary Figure S1).

### Quantification of the frequency of prophage excision

The frequency of prophage excision under different conditions was quantified by quantitative PCR. The number of total chromosomes was quantified based on the single-copy reference gene *gyrB.* The number of chromosomes devoid of each prophage was quantified using primers flanking each prophage (Supplementary Table S4), which only results in PCR products when the prophage is removed. The binding efficiency of the primer used to quantify the CP4So excision rate was tested with varying proportions of genomic DNA from wild-type versus genomic DNA from ΔCP4So (ranging from 1/10 to 1/1 000 000) as a template to generate a standard curve; the validity of this application for quantification was confirmed (Supplementary Figure S10).

### Detection of CP4So excision

To determine prophage excision at cold temperatures, *S. oneidensis*, Δ*alpA* or Δ*hns* cells were cultured at 30 °C until the turbidity at 620 nm reached 1.0 and then transferred to 15 °C or 4 °C. The cells were collected at different time points and used for quantification of the prophage excision rate (Figure 2 and Supplementary Figure S2). To determine CP4So excision during temperature shifts, *S. oneidensis* was cultured continuously during four temperature transitions. First, *S. oneidensis* cells were grown at 30 °C until the turbidity at 620 nm reached 1.0 and then transferred to 15 °C. At the end of 5 days at 15 °C, 1/100 cells were inoculated into fresh Luria–Bertani (LB) medium and transferred to 4 °C. Similarly, at the end of 5 days at 4 °C, 1/100 cells were inoculated into fresh LB medium and transferred back to 15 °C. At the end of 5 days at 15 °C, 1/100 cells were inoculated into fresh LB medium and transferred back to 30 °C. The cells were collected at the end of 5 days after each temperature shift and DNA was extracted to assess prophage excision by quantitative PCR.

### Construction of strains and plasmids

The strains and plasmids used in this study are listed in Table 1. Experiments were conducted at 30 °C for *S. oneidensis* and at 37 °C for *E. coli* in LB medium, unless otherwise noted. For culturing the *E. coli* WM3064 strain, 2,6-diamino-pimelic acid was added to 0.3 mM. In-frame deletions of single *S. oneidensis* genes were constructed by the Fusion PCR method using suicide plasmid pHGM01 (Jin *et al.*, 2013). Complementation of SsrA, AlpA, IntA and variant SsrA in *S. oneidensis* was conducted using the inducible expression plasmid pHGE (Shi *et al.*, 2013). After verification by DNA sequencing, the expression plasmids were conjugated into different *S. oneidensis* hosts. Plasmid pET28b-*hns* was constructed by following a previously described procedure (Liu *et al.*, 2015).

### Stress assay

Cells of wild-type, Δ*ssrA* and ΔCP4So strains were grown to a turbidity of 1.0 at 620 nm and diluted by 10^1^–10^6^ with a 0.85% NaCl solution via 10-fold serial dilution steps. The diluted cells were plated on LB agar containing different concentrations of gentamicin, kanamycin and streptomycin, to determine cell viability (Donegan *et al.*, 1991).

### Biofilm assay

Attached biofilm formation was assayed in 96-well polystyrene plates (Corning Costar, Cambridge, MA, USA) with crystal violet staining as described previously (Pratt and Kolter, 1998). To remove growth effects, biofilm formation was normalized by dividing the total biofilm by the maximal bacterial growth, as measured by turbidity at 620 nm for each strain. The assay was repeated at least three times independently.

### Light and fluorescence microscopy observation

The viability of cells cultured at 4 °C was tested using LIVE/DEAD BacLight Kit (Thermo Fisher Scientific, Waltham, MA, USA), which contains SYTO 9 and propidium iodide to differentiate between cells with intact membranes (live) and those with damaged membranes (dead). The cells were stained with 0.15 mM propidium iodine and 0.025 mM SYTO 9 dye for 20 min at ambient temperature. An aliquot of each cell suspension was also treated with 70% isopropyl alcohol to use as a dead cell control. The cells were imaged using a fluorescence microscope (Carl Zeiss, Jena, Germany).

### Quantitative real-time reverse-transcription PCR

Cells were collected at 30 °C or 4 °C at a turbidity of 1.0 at 620 nm. Total RNA was isolated as described previously (Ren *et al.*, 2004) and was used as the template for the quantitative real-time reverse-transcription PCR (qRT-PCR) reaction using the SuperScript III Platinum SYBR Green One-Step qRT-PCR Kit (Invitrogen, Carlsbad, CA, USA). The level of *rrsE* transcript was used to normalize the gene expression data. Primers for qRT-PCR are listed in Supplementary Table S4.

### Purification of H-NS

H-NS with six histidines at the C-terminus was purified via *E. coli* BL21(DE3)/pET28b. *E. coli* BL21(DE3)/pET28b-*hns* was grown in 500 ml LB medium with kanamycin and was induced with 1 mM isopropyl β-D-1-thiogalactopyranoside (IPTG) at turbidity of 0.5 at 620 nm for 5 h at 37 °C. Cells were collected and resuspended in 10 ml lysis buffer (50 mM potassium phosphate buffer pH 8.0, 500 mM NaCl and 1 mM phenylmethylsulfonyl fluoride) and then disrupted twice by a French Press (Thermo Fisher Scientific). Ni-NTA resin (Qiagen, Duesseldorf, Germany) was used according to the manufacturer's protocol. Purified protein was dialyzed against buffer (50 mM potassium phosphate buffer pH 7.6 and 300 mM KCl) twice at 4 °C overnight and the protein concentration was measured by using a Pierce BCA assay kit (Thermo Fisher Scientific).

### Electrophoretic mobility shift assays

Electrophoretic mobility shift assays were performed as described previously (Liu *et al.*, 2015). The promoter region of the *alpA* operon (P*_alpA_*) and *SO_3012* operon (P*_SO_3012_*) was amplified by PCR from genomic DNA with primer pairs alpA promoter-f/-r and SO_3012 promoter-f/-r (Supplementary Table S4). PCR products were purified and labeled with biotin using the Biotin 3′-end DNA Labeling Kit (Thermo Fisher Scientific). For binding reactions, biotin-P*_alpA_*/P*_SO_3012_* was incubated with purified H-NS protein either with or without unlabeled P*_alpA_*/P*_SO_3012_* probe, respectively, for 1 h at different temperatures.

## Results

### A mosaic prophage CP4So inserted in *ssrA*

**Figure 1:**
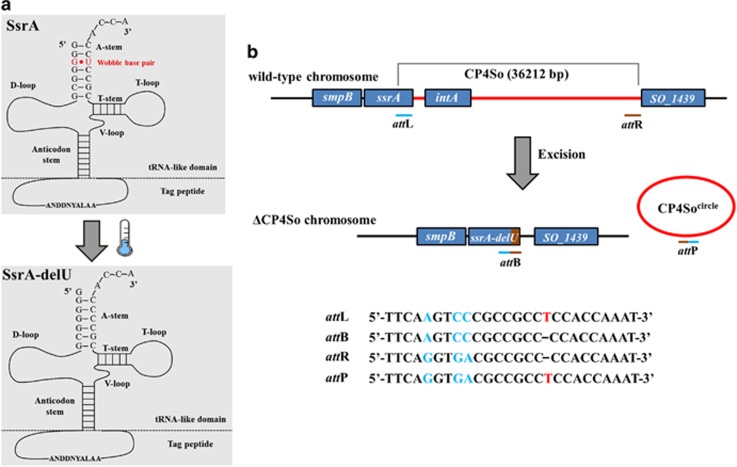
Prophage CP4So inserted in *ssrA*. (**a**) The structure of 3′-end of the wild-type SsrA and the mutated SsrA (SsrA-delU) is predicted by ARAGORN. Mutated SsrA lacks the wobble base pair (G·U) at the tRNA-like domain of the 3′-end. (**b**) Prophage CP4So inserted in *ssrA* was identified by the presence of P4-like integrase gene and two homologous attachment sites. Site-specific recombination occurs through the cross-over between *att*L and *att*R sites and generate ΔCP4So and phage-like circle CP4So_circle_, which is lost subsequently. Genes neighboring prophage CP4So are shown in blue. The *att*L attachment site within the *ssrA* gene is indicated as blue bar and the *att*R attachment indicated as brown bar. DNA sequences of the *att*P, *att*B, *att*L and *att*R are shown below. The deletion of a ‘T' at position 349 in *ssrA* is predicted due to site-specific recombination of *att*L and *att*R during prophage excision.

To explore the genetic changes that occur at cold temperatures, we performed whole-genome deep-sequencing using *Shewanella oneidensis* cells collected after growth at 4 °C and 30 °C. As shown in Table 2, a total of 10 mutations were identified at frequencies of 1–24% between the two samples, including 5 synonymous and 4 non-synonymous point mutations. Interestingly, we also identified a single-nucleotide deletion in the *ssrA* gene. The gene product of *ssrA* is a tmRNA, which monitors protein synthesis quality control and rescues stalled ribosomes in a process called *trans*-translation, which is required for normal bacterial growth (Komine *et al.*, 1994; Abo *et al.*, 2002). A prominent feature of all tmRNAs is a conserved tRNA-like domain at the 3′-end that contains a conservative G·U wobble base pair (Hou and Schimmel, 1988). Cold temperature resulted in deletion of a U at the 3′-end of SsrA, destroying this G·U wobble base pairing (Figure 1a). The *ssrA* gene is both a target for mobile DNAs and a passenger on others (Williams, 2003). Previous studies in other bacteria have indicated that prophage excision could lead to potential changes in SsrA (Rajanna *et al.*, 2003; Wang *et al.*, 2009). In *S. oneidensis*, an integrase gene, *intA* (*SO_1471*), is located at the 3′-end of the *ssrA* gene, which suggests the presence of a prophage element in SsrA. Indeed, we found a putative 36 kb mosaic prophage element inserted in SsrA. This element carries 30 protein-coding genes from *SO_1440* to *intA* and includes genes that encode integrases, restriction–modification systems, toxin–antitoxin systems, transposases, phage repressors and proteins with unknown functions (Figure 1b and Supplementary Table S1). There are two integrase genes present and the one near *ssrA* shares high similarity with the integrase P4-like prophage gene in *E. coli*; thus, we propose naming it prophage CP4So (for cryptic P4-like prophage in *S. oneidensis*). By comparing the two attachment sites of CP4So, we determined that excision of CP4So from the host genome could lead to the deletion of a U at the 3′-end of SsrA due to a site-specific recombination event (Figure 1b). Accordingly, we hypothesized that excision of the CP4So prophage occurs at cold temperatures, leading to deletion of the U in SsrA.

### Prophage CP4So excises at cold temperatures

To test this hypothesis, a PCR-based assay was first performed to screen for prophage-excised colonies at a cold temperature; excision of the P4-like prophage through a site-specific recombination event between the *attL* and *attR* sites should completely remove the phage from the host chromosome (Sozhamannan *et al.*, 2006). From 200 colonies randomly collected at 4 °C after grown for 7 days, we obtained two positive colonies that only generated the expected PCR products after CP4So had excised (Supplementary Figure S1). DNA sequencing of these positive colonies confirmed the complete removal of 36 kb from the host genome and deletion of a U at the 3′-end of SsrA was found for the prophage-excised cells (called ΔCP4So). Thus, we demonstrate that the CP4So prophage excised at a cold temperature.

**Figure 2:**
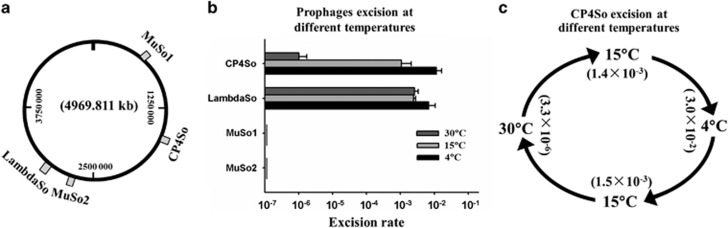
Prophage CP4So only excises at cold temperatures. (**a**) The location of the four identified prophages in *S. oneidensis*. (**b**) The excision of four prophages in the wild-type strain was measured after 3 days culturing at different temperatures. (**c**) Schematic depiction of changes of CP4So excision rate at different temperatures. Excision of CP4So in the wild-type strain was measured after 5 days in each temperature shift and 1/100 inoculum was used at each transition. Data are from three independent cultures and one s.d. is shown in **b** and **c**.

Next, we tested the effects of temperature on prophage excision using a quantitative PCR-based assay. In addition to CP4So, three other prophages (LambdaSo, MuSo1 and MuSo2) have also been found in the *S. oneidensis* genome (Figure 2a). When grown at warm temperatures of 30 °C or 25 °C, the excision rates of CP4So remained at very low levels throughout the growth phase (~1 out of 10^6^ cells), but these rates gradually increased after a lag time when exposed to low temperatures (Supplementary Figure S2), reaching ~1 out of 10^3^ at 15 °C and 1 out of 10^2^ cells at 4 °C after 3 days (Figure 2b and Supplementary Figure S2). Furthermore, we tested how temperature fluctuation during seasonal change affects prophage excision. When downshifted from 30 °C to 15 °C, the excision rates increased by ~420-fold from 3.3 × 10^−6^ to 0.14% when further downshifted from 15 °C to 4 °C, the excision rates increased by another 20-fold, reaching up to 3.0%. In addition, when upshifted from 4 °C to 15 °C, the excision rate returned to 0.15% and then back to 3.3 × 10^−6^ when further shifted from 15 °C back to 30 °C (Figure 2c). In contrast, the three other prophages, LambdaSo, MuSo1 and MuSo2, were relatively stable during the temperature shifts (Figure 2b). Unlike prophage LambdaSo, CP4So did not excise on initiation of the SOS response, which is known to trigger DNA damage (Supplementary Figure S3). Moreover, alteration in CP4So excision was not detected during oxidative stress (1 mM H_2_O_2_), kanamycin stress (2 μg ml^−1^), nutritional stress (grown in M9 minimal medium) or at different growth stages when tested at 30 °C. Taken together, these results show that the induction of CP4So excision is a specific stress response that only occurs at cold temperatures.

### tmRNA function is abolished on prophage excision

**Figure 3:**
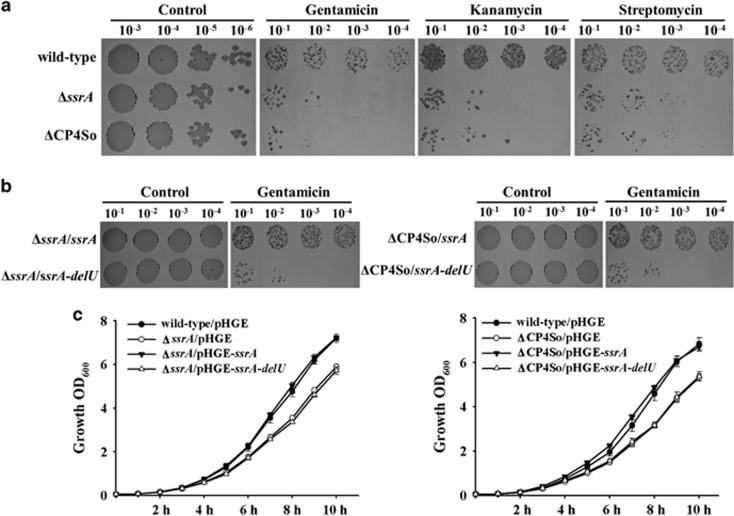
Function of tmRNA is abolished on prophage excision. (**a**) Survival of cells after exposure to sublethal concentrations of kanamycin (2.5 μg ml^−1^), gentamicin (1 μg ml^−1^) and streptomycin (10 μg ml^−1^) for the wild-type, Δ*ssrA* and ΔCP4So strains. (**b**) Survival of cells after exposure to sublethal concentrations of gentamicin (1 μg ml^−1^) for the wild-type, Δ*ssrA* and ΔCP4So strains expressing a full-length *ssrA* (pHGE-*ssrA*) or a mutated *ssrA* (pHGE-*ssrA*-*delU*). Data are from three independent cultures and only representative images are shown in **a** and **b**. (**c**) Growth of wild-type, Δ*ssrA* and ΔCP4So expressing a full-length *ssrA* (pHGE-*ssrA*) or a mutated *ssrA* (pHGE-*ssrA*-*delU*) in LB medium. Data are from three independent cultures and one s.d. is shown.

To test whether the single deletion of a U caused by CP4So excision affects SsrA function *in vivo*, we first compared the physiological changes caused by such excision with those caused by removal of the full-length *ssrA* gene. The *ssrA* deletion mutant displayed increased susceptibility to the miscoding antibiotic gentamicin compared with the wild-type strain when tested at a sub-lethal concentration (1 μg ml^−1^) at 30 °C (Figure 3a). However, at higher concentrations (for example, 10 μg ml^−1^), gentamicin inhibited the growth of both the wild-type cells and Δ*ssrA* cells to the same extent (results not shown). These results were expected, because it has been reported that SsrA in *E. coli* contributes to the survival of cells exposed to miscoding antibiotics, including gentamicin, but only at sublethal concentrations (Abo *et al.*, 2002). Similar results were obtained when the miscoding antibiotics streptomycin and kanamycin were tested, with reduced Δ*ssrA* survival observed at sublethal concentrations (Figure 3a). With 1 μg ml^−1^ gentamicin treatment, the ΔCP4So strain showed increased susceptibility compared with the wild-type strain, but this was comparable to the Δ*ssrA* strain (Figure 3a), suggesting that CP4So excision disrupted the quality control function of SsrA in protein synthesis.

Furthermore, two plasmids for expression of the wild-type (pHGE-*ssrA*) and variant (without the U at position 349, pHGE-*ssrA-delU*) SsrA were constructed and used to complement Δ*ssrA* and ΔCP4So, after which resistance to gentamicin was tested. Ectopic expression of wild-type SsrA restored resistance to sublethal concentrations of gentamicin in Δ*ssrA* and ΔCP4So hosts, whereas expression of the variant SsrA failed to restore the wild-type phenotype (Figure 3b). Similar results were obtained when sublethal concentrations of streptomycin and kanamycin were tested, that is, expression of wild-type SsrA but not the variant restored survival in the Δ*ssrA* and ΔCP4So strains (data not shown). In addition, growth of the ΔCP4So and Δ*ssrA* strains was reduced to a similar level at 30 °C in LB medium (Figure 3c) and expression of wild-type SsrA but not the variant restored growth in the Δ*ssrA* and ΔCP4So strains (Figure 3c). Therefore, the U at 349 position of SsrA is crucial for its activity at warm temperatures, and CP4So excision abolishes the function of SsrA by removing this crucial base. Moreover, although a functional SsrA was essential for bacterial survival in the presence of sublethal concentrations of antibiotics at warm temperatures, Δ*ssrA* and ΔCP4So displayed the same phenotype as the wild-type strain at 4 °C, suggesting that the function of *ssrA* in quality control of protein synthesis was not required at cold temperatures (Supplementary Figure S4).

### Excision of CP4So enhances biofilm formation at cold temperatures

**Figure 4:**
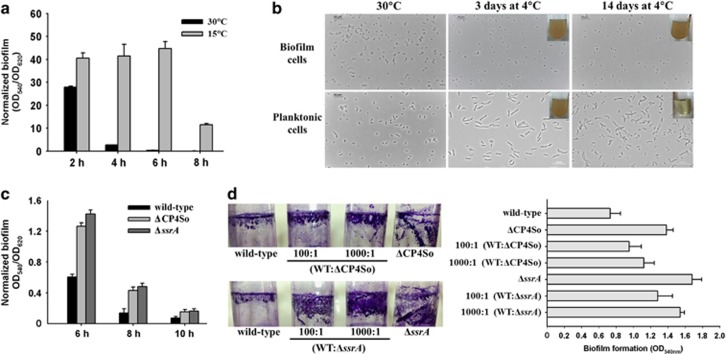
Excision of CP4So enhances biofilm formation at cold temperatures. (**a**) Attached biofilm formation of the wild-type strain at 30 °C and 15 °C, respectively. (**b**) Morphology of the biofilm cells and planktonic cells at 30 °C and 4 °C. The image in the upper right corner of each panel shows the turbidity of cultures after transferring these cells to grow at 30 °C with fresh LB medium for 48 h. (**c**) Attached biofilm formation of the wild-type, ΔCP4So and Δ*ssrA* strains at 30 °C. (**d**) Attached biofilm formation of mixed population of the wild-type, ΔCP4So and Δ*ssrA* strains at 15 °C shown by crystal violet staining in the glass tube (left panel) and quantified in 96-well polystyrene plate (right panel). Data are from three independent cultures. One s.d. is shown in **a**, **c** and **d**, and representative images were shown in **b** and **d**.

Surface-attached biofilm formation appears to be promoted by non-optimal growth conditions in many bacterial species; hence, we first assessed biofilm formation of *S. oneidensis* at different temperatures. *S. oneidensis* formed more attached biofilms at 15 °C than at an optimal growth temperature of 30 °C, increasing by 15.6±2.0-fold at 4 h, 116.1±9.5-fold at 6 h and 81.1±5.2-fold at 8 h. These results suggest that cold temperatures promote an attachment-associated lifestyle (Figure 4a). With biofilm formation at 15 °C, *S*. *oneidensis* cells remained viable for more than 3 months after exposure to 4 °C (data not shown), whereas planktonic cells survived no longer than 14 days at this temperature. Microscopic images showed that the majority of the planktonically growing cells became elongated after 3 days at 4 °C and lysed after 14 days (Figure 4b and Supplementary Figure S5); in contrast, the biofilm cells were not elongated after the same incubation time at 4 °C (Figure 4b). As CP4So was induced to excise at low temperatures, we tested how excision affects biofilm formation. The ΔCP4So and Δ*ssrA* strains both formed more attached biofilms at 30 °C than the wild-type strain at early time points (Figure 4c). Next, we investigated the effects on biofilm formation of a sub-population of mutated cells in the wild-type population by combining wild-type cells with the two mutated strains at two different proportions to produce four mixed populations. Compared with the wild-type population, the mixed populations all formed a greater amount of attached biofilms on both a glass surface (Figure 4d, left panel) and a polystyrene surface (Figure 4d, right panel) at 15 °C, indicating that the presence of a relatively small population of prophage-free cells or *ssrA*-deficient cells is able to promote biofilm formation by the entire population.

To explore whether CP4So excision contributes to cold adaptation, survival of the wild-type strain and ΔCP4So strain was investigated at 4 °C. Live/dead staining showed that both strains were able to survive for 12 days. Very few wild-type cells were found to be viable after 14 days (<1%), with a higher percentage of ΔCP4So cells found to be viable (>10%) (Supplementary Figure S5b). When transferred to a warm temperature of 30 °C, cells of the ΔCP4So strain had resumed growth after 14 days but not those of the wild-type strain; similar results were obtained after 16 days. In addition, similar to the ΔCP4So strain, the Δ*ssrA* strain also showed higher survival than wild-type, which suggests that disrupting SsrA increases survival under cold conditions (Supplementary Figure S5).

### H-NS represses CP4So excision through the excisionase gene *alpA*

**Figure 5:**
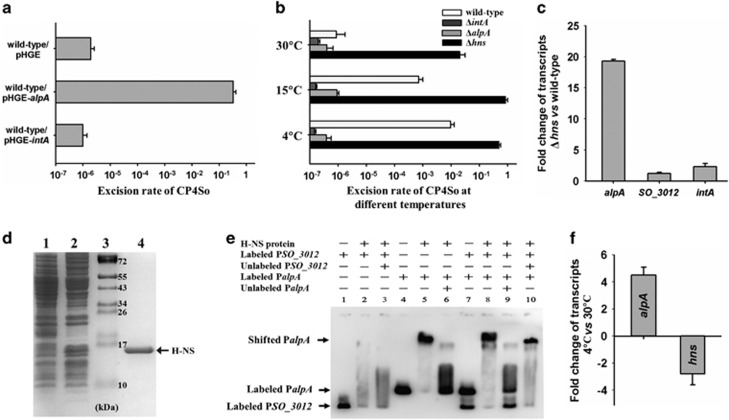
H-NS represses CP4So excision through *alpA*. (**a**) Excision of CP4So was induced by overproduction of excisionase AlpA in the wild-type strain. (**b**) Excision of CP4So in the wild type, Δ*intA*, Δ*alpA* and Δ*hns* strains was measured after 3 days culturing at different temperatures. (**c**) The fold change of the *alpA*, *SO_3012* and *intA* transcription level obtained by comparing the Δ*hns* with the wild-type strain. (**d**) Purification of H-NS protein. Strain *E. coli* BL21(DE3)/pET28b-*hns* was used to overexpress H-NS protein. Protein expression was induced with 1 mM IPTG (lane 2). The untreated cells were used as a negative control (lane 1). Proteins in the bacterial cell lysates were separated on SDS-PAGE. The protein marker (Pageruler prestained protein ladder) was loaded in lane 3 and the purified H-NS protein in lane 4 was marked with an arrow. (**e**) Electrophoretic mobility shift assay (EMSA) shows that H-NS binds to the promoter of *alpA* but not to the promoter of *SO_3012*. (**f**) The fold change of the *alpA* and *hns* transcription level at 4 °C versus at 30 °C in the wild-type strain. Data are from three independent cultures and one s.d. is shown in **a**, **b**, **c** and **f**.

Gene *SO_4821* encoding putative excisionase AlpA is harbored on prophage CP4So and the sequence exhibits 47% sequence identity with AlpA of *E. coli* K-12 prophage CP4-57 (Supplementary Figure S6). As quantified by quantitative PCR, AlpA overproduction caused excision in a high proportion of *S. oneidensis* cells (33±8%), whereas IntA overproduction did not induce CP4So excision at 30 °C (Figure 5a). Furthermore, no CP4So excision was detected for the *alpA* and *intA* deletion mutants at 15°C or 4 °C (Figure 5b), which indicated that AlpA functions as an excisionase and is required for CP4So excision.

The histone-like DNA-binding protein H-NS is known to respond to temperature change and exerts transcriptional control over temperature-regulated genes, especially horizontally transferred genes (Lateana *et al.*, 1991; Ono *et al.*, 2005), and the gene encoding H-NS in *S. oneidensis* is *SO_3146*. Among the four prophages, CP4So is specifically silenced by H-NS at 30 °C, as the excision rate is greatly increased (5400±156-fold) when *hns* is deleted (Figure 5b). In contrast, no change in excision for prophages MuSo1 or MuSo2 was observed, with the excision rate of LambdaSo increasing only slightly (5.1±0.4-fold), in the absence of *hns*. Bioinformatic analysis revealed two H-NS binding sites (5′-GATAATG-3′) in the promoter region of *alpA*, whereas none are present in the promoter of the LambdaSo excisionase gene *SO_3012* (Supplementary Figure S7). To further explore how H-NS controls AlpA to regulate CP4So excision, transcription of *alpA* was tested in the Δ*hns* strain by qRT-PCR. H-NS specifically repressed *alpA* transcription (19.3±0.3-fold) but not *SO_3012* nor *intA* at 30 °C (Figure 5c). We also performed an electrophoretic mobility shift assay using purified H-NS protein, to further examine the role of H-NS in regulating *alpA* (Figure 5d). As shown in Figure 5e, H-NS bound and shifted the promoter of *alpA* (lanes 4–6) but did not demonstrate specific binding to the LambdaSo excisionase gene *SO_3012* (lanes 1–3). We also showed that in the presence of both promoters, H-NS selectively bound and shifted the *alpA* promoter (lanes 7–10). Thus, the slight increase in LambdaSo excision in the Δ*hns* strain is more likely to be due to an indirect effect between the H-NS protein and LambdaSo. Furthermore, along with the increase in CP4So excision that accompanies a temperature downshift, *hn*s transcription was repressed (−2.8±0.8-fold) but that of *alpA* induced (4.5±0.6-fold) when the temperature was downshifted from 30 °C to 4 °C (Figure 5f). Because of the reduced expression of *hns* at 4 °C compared with 30 °C, repression of CP4So excision by H-NS was also greatly reduced from 5400±156-fold at 30 °C to 24.5±3.3-fold at 4 °C (Figure 5b). It is noteworthy that the binding of H-NS protein to the promoter sequence of *alpA* was not affected by the temperature (Supplementary Figure S8). Collectively, these experiments demonstrate that H-NS specifically represses CP4So excision via direct binding to the promoter of *alpA* at warm temperatures, and that this repression is reduced at cold temperatures due to reduced expression of H-NS.

## Discussion

**Figure 6:**
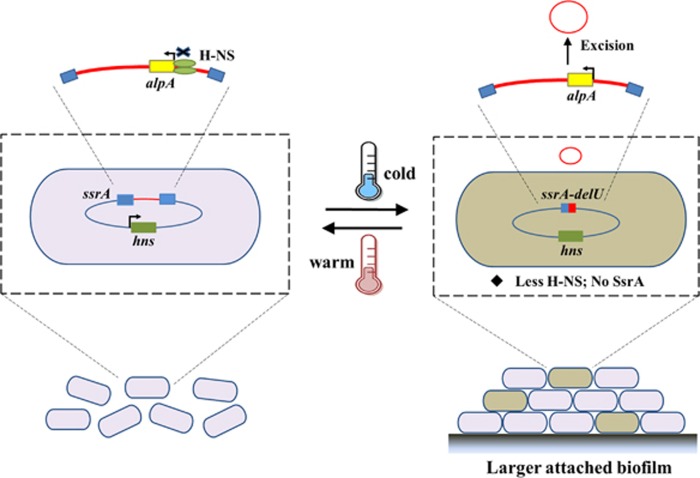
Proposed mechanism of cold adaptation regulated by prophage excision. The stable residence of CP4So in host genome is preferred at warm temperature, which ensures the function of tmRNA. H-NS silences CP4So excision by binding to the promoter of *alpA* and represses expression of *alpA* at warm temperature. Although at cold temperatures, due to the de-repression of H-NS to *alpA*, CP4So excises and the excision leads to the formation of subpopulation (0.1–3%) of prophage-free cells with increased attached biofilm formation for the entire population.

Temperature is a ubiquitous environmental stress for bacteria and their survival depends on the initiation of appropriate responses to the cellular stress induced by drastic temperature shifts. In psychrotrophic bacteria, physiological changes in the cell membrane, protein, RNA and DNA occur, ensuring survival at non-optimal cold temperatures. In this study, we used whole-genome deep-sequencing to probe changes in genetic variability in a psychrotrophic bacterium, *S. oneidensis*, during winter versus summer. A total of 10 mutations with different frequencies at the population level were found. Examining one tmRNA gene mutation in the cold sample revealed a new function for a cryptic prophage in the cold tolerance of this bacterium. In *S. oneidensis*, prophage CP4So is integrated in the tmRNA gene and the element remains stable at warm temperatures and on initiation of the SOS response. However, when the temperature decreases, CP4So is induced to excise, which results in a subpopulation of prophage-free cells. Two direct consequences of CP4So excision on host physiology include disruption of tmRNA function and removal of 30 prophage genes from the host genome. Although the function of tmRNA in quality control of protein synthesis was not required at cold temperatures (Supplementary Figure S4), disruption of tmRNA function did increase attached biofilm formation and cell survival at cold temperatures. Conversely, tmRNA function was found to be critical for maintaining proper protein synthesis at warm temperatures. Collectively, as illustrated in Figure 6, these results suggest that the stable residence of CP4So is preferred at warm temperatures to ensure a functional tmRNA, whereas at cold temperatures CP4So excision leads to formation of 0.1–3% prophage-free cells with increases in attached biofilm formation. As an attached biofilm is critical for prolonged survival at cold temperatures (Tribelli and Lopez, 2011; Zhang *et al.*, 2015), prophage CP4So functions as an important regulatory switch during temperature shifts.

In addition to tmRNA, five synonymous and four non-synonymous point mutations were identified. Two (SO_2545 and SO_4717) of the latter belong to the histidine kinase family (Table 2), members of which acts as membrane-associated sensors and, along with a cytoplasmic response regulator, can act as temperature sensors (Sengupta and Garrity, 2013). In particular, it has been reported that the CroS and VirA sensor kinases in *Pseudomonas syringae* and *Agrobacterium tumefaciens* are critical for host virulence on temperature upshift or downshift (Banta *et al.*, 1998; Braun *et al.*, 2007). In addition, the PhoQ sensor kinase in *Edwardsiella tarda* detects alterations in temperature via a conformational change in its secondary structure, thereby activating type III and type IV secretion systems (Chakraborty *et al.*, 2010). However, further investigation is required to determine whether the histidine kinases SO_2545 and SO_4717 are cold-shock kinase sensor proteins. In addition, one of the non-synonymous point mutations (SO_3735) encodes a peptidoglycan glycosyltransferase and participates in peptidoglycan biosynthesis, which forms a protective shell around bacterial cell membranes. It also has been reported that peptidoglycan glycosyltransferase is involved in cell elongation and cell division (Macheboeuf *et al.*, 2006). The roles of peptidoglycan glycosyltransferase gene in cold environment need further investigation.

For marine phages, lysogeny is commonly regarded as a beneficial state, as it promotes the propagation of the prophage and its host in conditions unfavorable for rapid growth (Paul, 2008). Metagenomic and comparative analysis suggested that in polar regions, lysogenic conversion of prophages to lytic cycle increased in the summer in responses to increased bacterial abundance and productivity, but poor host physiology driving prophage toward lysogeny in the spring (Brum *et al.*, 2016). The prevalence of lysogeny was also found in the cold environment (Angly *et al.*, 2006) and the induction of prophage is high during the warmer seasons (Cochran and Paul 1998). These studies demonstrated the importance of active prophages in controlling bacterial abundance and diversity via lysis the host. However, cryptic prophages are captured in the bacterial chromosome and they do not form active phage particles or lyse their captors on induction. Although the lysogenic conversion of active and cryptic prophages could be both affected by the physiological state of the host, environmental and/or host factors trigger prophage induction and the outcome of prophage induction could be different. Active prophage excision is normally induced by mitomycin C, which triggers the SOS response and generates DNA damage, yet many cryptic prophages remain stable on SOS response. For example, when originally isolated, *E. coli* K-12 harbors an active prophage Lambda, which is inducible on SOS responses (Weigle and Delbruck, 1951), but eight out of nine cryptic prophages remain stable in the host on SOS responses (Wang *et al.*, 2010). Previous genome analysis revealed three prophage elements in *S. oneidensis*: one lambda-like phage LambdaSo and two Mu-like prophages (Heidelberg *et al.*, 2002). LambdaSo is capable of forming infectious phage particles and the initiation of the SOS response can induce the lysogenic conversion of LambdaSo (Godeke *et al.*, 2011; Binnenkade *et al.*, 2014). In contrast, CP4So did not respond to SOS responses and remain stable in the host genome during DNA damage. Recent studies have revealed that prophage excision under specific conditions can also affect host physiology without triggering lytic production (Feiner *et al.*, 2015). Cryptic prophage excision during biofilm formation or host invasion in *E. coli* (Wang *et al.*, 2009) and *L. monocytogenes* (Rabinovich *et al.*, 2012) can create genetic or phenotypic variation at the population level by generating a sub-population of prophage-free cells, which may benefit the population as a whole. Here we show that CP4So in *S. oneidensis* was induced to excise at cold temperatures, resulting in a subpopulation of prophage-free cells showing increased attached biofilm formation and thus increased fitness at cold temperatures. These results indicate that the stability of the cryptic prophage is tightly coupled with the physiology of the host cell, and that bacteria can respond to stress in various ways that involve editing their genomes through prophage excision. SsrA houses a remarkable diversity of genetic elements in various bacteria and integration of those genetic elements were usually found at the conserved tRNA-like structure at the 3′-end of ssrA (Williams, 2003). Indeed, it is an open question whether prophage inserted in SsrA or other critical genes is involved with host physiology and adaptive processes in many other environmental and pathogenic bacteria.

We also provide evidence that the host factor H-NS controls CP4So excision in *S. oneidensis* by regulating *alpA* expression. Through qRT-PCR and electrophoretic mobility shift assays, we showed that H-NS strongly represses CP4So excision but does not control excision of the prophage LambdaSo. In contrast, although the *alpA* gene in the CP4-like prophage shares high similarity with *S. oneidensis* CP4So and *E. coli* K-12 CP4-57, H-NS repression does not regulate CP4-57 excision in *E. coli* (Hong *et al.*, 2010); instead, another DNA-binding protein, Hha, induces CP4-57 excision (Wang *et al.*, 2009). In addition, prophage CP4-57 in mesophilic *E. coli* K-12 did not induce excision at cold temperatures (data not shown). Thus, the selective silencing of prophage excision by H-NS is both species specific and prophage specific.

In addition to the direct binding of H-NS to the *alpA* promoter, another important role for H-NS is as a thermal sensor controlling compaction of the nucleoid, as suggested by the observation that the nucleoid undergoes a dramatic condensation in H-NS-overproducing cells (Amit *et al.*, 2003). In *E. coli* and *Salmonella*, H-NS accumulates during the cold shock response (Jones *et al.*, 1987; Ono *et al.*, 2005) and the cold shock protein CspA, which regulates DNA supercoiling at low temperatures, is involved in enhancing *hns* transcription in *E. coli* (Lateana *et al.*, 1991). A supercoiled DNA template was found to be essential for *in vitro* transcription of a specific gene at low temperatures (Uma *et al.*, 1999). Rather than H-NS accumulation at cold temperatures, we show that H-NS transcription is repressed in *S. oneidensis*; H-NS was also previously reported as one of 17 proteins significantly repressed in *S. oneidensis* at 3 °C in comparison with 22 °C (Abboud *et al.*, 2005). Although our *in vivo* studies showed that H-NS reduced repression of *alpA* expression at cold temperatures, our electrophoretic mobility shift assay results showed that H-NS can still bind to the promoter of the *alpA* gene *in vitro* at cold temperatures of 4 °C or 15 °C (Supplementary Figure S8). Thus, it remains unresolved whether H-NS in psychrotrophic bacteria directly controls *alpA* expression by binding to the promoter to prevent RNA polymerase binding or by altering DNA topology *in vivo* in response to a temperature shift.

The composition of bacterial genomes can be modified through the acquisition of mobile elements. In this study, we found that *ssrA* harbors various mobile elements in different *Shewanella* spp. isolated from different locations (Supplementary Figure S9). Although a large proportion of genes in these mobile elements remain to be determined, restriction–modification systems and endonucleases are commonly found in elements residing in the *ssrA* gene of different *Shewanella* species and the function of these mobile genes has been associated with phage exclusion and pathogenicity. Using large-scale gene–phenotype maps for *S. oneidensis*, a previous study showed that inactivation of the *SO_1458* and *SO_1459* genes of CP4So significantly reduced the swimming motility of *S. oneidensis*, which is important for nutrient acquisition in the aquatic environment (Deutschbauer *et al.*, 2011). Mass spectrometry coupled with the accurate mass and time tag method has identified SO_1459 as the only outer membrane protein that is overproduced after Fe (III) exposure, which indicates the importance of the ability of *S. oneidensis* to reduce Fe (III) under anoxic conditions (Elias *et al.*, 2005). In addition, using a genome-wide fitness profiling assay, Deutschbauer *et al.* (2011) demonstrated that inactivation of SO_1471 is able to increase the sensitivity to putrescine when it serves as the sole nitrogen source and the ability to use putrescine is important for *Shewanella* spp. as components of nitrogen geochemical cycling in the ocean (Brettar *et al.*, 2002). Moreover, the roles of bacterial toxin/antitoxin systems in biofilm formation (Ren *et al.*, 2004; Kim *et al.*, 2008), persistent cell formation and phage inhibition (Pecota and Wood, 1996; Hazan and Engelberg-Kulka, 2004) are becoming increasingly clear. Indeed, toxin/antitoxin systems in *E. coli* prophages have been linked to persistence or antibiotic resistance, including the *relE* toxin gene of prophage Qin, the *yeeV* toxin gene of prophage CP4-44 (Shah *et al.*, 2006) and the *ralR* toxin gene of prophage rac (Guo *et al.*, 2014). Four toxin/antitoxin genes are present in CP4So (*SO_1444*, *SO_1445*, *SO_1440* and *SO_1453*) and we are currently investigating the functions of these genes.

Important questions regarding bacteria–phage interactions remain, such as why and how do bacteria retain prophage DNA? In the cases of the cryptic prophages CP4So in *S. oneidensis* and CP4-57 in *E. coli* K-12, both regions are subjected to massive loss of prophage DNAs; nonetheless, removal of these prophages is inhibited by host factors, ensuring the relatively stable residence of these genes in the host genome under normal growth conditions. However, occasional loss of these prophages is observed under specific conditions, such as during biofilm formation in *E. coli* K-12 and during cold growth in *S. oneidensis*, suggesting that despite being costly, a strategy of losing specific prophage genes might be adopted in a specific environment to enhance fitness in social groups. Increasing evidence reveals the important functions of individual prophage genes within the context of host physiology, especially during conditions of stress. By applying whole-genome sequencing, an increasing number of prophages, such as DNA fossils, will be identified in bacterial genomes through large-scale phenotypic array analysis and additional functions for prophage DNA will be revealed under different conditions, which will help provide fundamental insight into the evolution of bacterial genomes.

## Figures and Tables

**Table 1:** Bacterial strains and plasmids

*Strains and plasmids*	*Genotype/relevant characteristics*	*Source*
S. oneidensis *strains*		
Wild-type	MR-1	ATCC 700550
ΔCP4So	The whole CP4So excised in MR-1	This study
Δ*ssrA*	Full deletion of *ssrA* gene in MR-1	This study
Δ*hns*	Full deletion of *hns* gene in MR-1	This study
Δ*alpA*	Full deletion of *alpA* gene in MR-1	This study
Δ*intA*	Full deletion of *intA* gene in MR-1	This study

E. coli *strains*		
K-12 BW25113	*lacI*^q^ *rrnB*_T14_ Δ*lacZ*_WJ16_ *hsdR*514 Δ*araBAD*_AH33_ Δ*rhaBAD*_LD78_	(Baba *et al.*, 2006)
WM3064	*thrB*1004 *pro thi rpsL hsdS lacZ*ΔM15 RP4-1360Δ(*araBAD*)567 Δ*dapA*1341::[*erm pir(*wt)]	(Shi *et al.*, 2013)
BL21(DE3)	F^−^*ompT hsdS*_*B*_*(r*_*B*_^−^*m*_*B*_^−^*) gal dcm λ*(DE3) Ω P_*tacUV5*_::T7 polymerase	Novagen

*Plasmids*		
pHGE	pHGE-P_*tac*_, Km^R^, IPTG-inducible expression plasmid	(Shi *et al.*, 2013)
pHGE-*ssrA*	Km^R^; plasmid for expressing the wild-type *ssrA*	This study
pHGE-*ssrA*-*delU*	Km^R^; plasmid for expressing the mutated *ssrA*	This study
pHGE-*alpA*	Km^R^; expression plasmid for *alpA* of MR-1	This study
pHGE-*intA*	Km^R^; expression plasmid for *intA* of MR-1	This study
pHGM01	Gm^R^; Cm^R^; Ap^R^; *sacB*; Ori-R6K; suicide plasmid for generating in-frame deletions	(Jin *et al.*, 2013)
pET28b-*hns*	Km^R^, *lacI*^q^, pET28b P_*T7-lac*_:: *hns* with C-terminal His-tagged	This study

Abbreviation: IPTG, isopropyl β-D-1-thiogalactopyranoside.

Bacterial strains and plasmids used in this study. Kanamycin (50 μg ml^−1^) and gentamicin (15 μg ml^−1^) were used to maintain pHGE-based and pHGM01 plasmids, respectively.

**Table 2:** Mutations of the *S. oneidensis* at 4 °C identified by whole-genome deep-sequencing

*ID*	*GenBank ID*	*Gene product*	*Genome position*	*Change in DNA*	*Change in protein or RNA*	*Frequency*
**M1**	**SO_0673**	**Mu phage uncharacterized** **protein**	**691074**	**156 (T→G)**	**52 (I→M)**	**0.24**
**M2**	**SO_m003**	**tmRNA (SsrA)**	**1538151**	**349**	**Deletion of U**	**0.02**
**M3**	**SO_2545**	**Histidine kinase**	**2679697**	**669 (C→T)**	**224 (P→S)**	**0.12**
M4	SO_2545	Histidine kinase	2679698	669 (A→G)	223 (V→V)	0.12
M5	SO_2940	Lambda phage tail fiber protein J	3077206	3267 (G→A)	1089 (Q→Q)	0.19
M6	SO_2940	Lambda phage tail fiber protein J	3077215	3258 (C→A)	1086 (A→A)	0.21
M7	SO_2940	Lambda phage tail fiber protein J	3077236	3237 (A→C)	1079 (A→A)	0.17
**M8**	**SO_3735**	**Peptidoglycan transglycosylase**	**3879087**	**419 (C→A)**	**140 (A→E)**	**0.01**
M9	SO_3923	Fe hydrogenase maturation rSAM protein	4071260	1113 (T→C)	371 (G→G)	0.13
**M10**	**SO_4717**	**Tungstate-responsive histidine kinase**	**4921610**	**923 (C→G)**	**308 (S→C)**	**0.10**

Abbreviation: tmRNA, transfer-messenger RNA.

Bold type indicates the mutations that lead to the change in protein or RNA.
