# Supplementary material for: Cold adaptation regulated by cryptic prophage excision in Shewanella oneidensis
Source: ISME J. 2016 Aug 2;10(12):2787–800. doi: 10.1038/ismej.2016.85 (PMC5148205; doi:10.1038/ismej.2016.85)

SUPPLEMENTARY FILE

Cold Adaptation Regulated by Cryptic Prophage Excision in *Shewanella oneidensis*

Zhenshun Zeng[1], Xiaoxiao Liu[1], Jianyun Yao[1,2], Yunxue Guo[1], Baiyuan Li[1,2], Yangmei Li[1,2], Nianzhi Jiao[3,4], Xiaoxue Wang[1*]

[1]Key Laboratory of Tropical Marine Bio-resources and Ecology, Guangdong Key Laboratory of Marine Materia Medica, RNAM Center for Marine Microbiology, South China Sea Institute of Oceanology, Chinese Academy of Sciences, Guangzhou 510301, PR China

[2]University of Chinese Academy of Sciences, Beijing 100049, China

[3]State Key Laboratory of Marine Environmental Sciences, [4]Institute of Marine Microbes and Ecospheres, Xiamen University, China Xiamen University, China

*To whom correspondence should be addressed. E-mail: xxwang@scsio.ac.cn

Running head*:* Prophage excises at low temperature

Table S1. List of 30 CP4So genes and the neighboring gene *ssrA* (the last row) in *S. oneidensis*.

Gene	Start	Stop	Direction	Length(nt)	Gene function
SO_1440	1502474	1503232	P	759	Bifunctional toxin-antitoxin system HepN family
SO_1441	1503494	1504570	P	1077	Putative cytoplasmic protein
alpA	1504689	1504901	P	213	Phage lytic protein AlpA, SO_4821
SO_1442	1504940	1506409	P	1470	Hypothetical protein
SO_1443	1506725	1507402	P	678	unknown function DUF3296 YagK
SO_1444	1507549	1507842	N	294	Toxin module of RelE/StbE family
SO_1445	1507805	1508113	N	309	Antitoxin module of toxin-antitoxin
SO_1447	1508400	1509122	N	723	Retinol acyltransferase domain protein
SO_1448	1509202	1509768	N	567	phage integrase family
SO_1450	1509877	1510284	N	408	Protein of unknown function DUF7287
SO_1451	1510471	1510884	N	414	Protein of unknown function DUF2787
SO_1453	1511070	1511444	N	375	Antitoxin of the YpjF-YfjZ toxin-antitoxin
SO_1454	1511587	1511949	N	363	Hypothetical protein
SO_1455	1511900	1512463	N	474	DNA repair protein, RadC family
SO_1456	1512539	1513495	N	957	ISSod11 transposase TnpA_ISSod11
SO_1457	1514245	1516299	P	2055	Type II restriction-modification system, M subunit
SO_1458	1516296	1517591	P	1296	B-loop ATPase superfamily protein
SO_1459	1517594	1518256	P	663	Concanavalin A-like lectin/glucanase domain protein
SO_1460	1518253	1519662	P	1410	Type I restriction-modification system, S subunit
SO_1461	1519672	1523802	P	4131	Serine/threonine protein kinase
SO_1462	1523799	1525445	P	1647	Type I restriction-modification system locus
SO_1463	1525442	1532103	P	6662	Unknown
SO_1464	1530017	1531176	P	1160	ISSod1 transposase TnpA_ISSod1
SO_1467	1532409	1533017	P	609	Unknown
SO_1468	1533026	1534087	P	1062	ADP-ribose binding domain-containing protein
SO_1469	1534142	1534396	N	255	Transcriptional regulator Cro/CI family
SO_4773	1534619	1534951	P	333	Predicted membrane protein
SO_4774	1534987	1535256	N	270	Unknown
SO_1470	1535896	1536510	P	615	Hypothetical protein
intA	1536629	1537876	N	1248	P4-like prophage integrase IntA , *SO_1471*
SO_m003	1538145	1538499	N	355	Transfer-messenger RNA, tmRNA

Table S2. Statistical analysis of the whole-genome deep-sequencing results includes the average insertion length of library, total reads, Q20% and the average coverage with the reference genome.

Samples	Insert Size	Read Length	Raw Data (Mb)	Raw Reads Number	Q20 (%)	Average coverage	Clean Data (Mb)	Clean Reads Number
4 ℃	300	151	5751.7	19045375*2	95.7	1157x	5472.8	18245631*2
30 ℃	300	151	2105.8	6972888*2	95.3	423x	1990.8	6632972*2

Table S3. The alignment of the whole-genome deep-sequencing data was shown using the genome of *S. oneidensis* MR-1 (5 Mb, http://www.ncbi.nlm.nih.gov/genome/?term=MR-1) as reference.

Samples	PCR-dup Rate	Mapped Data (Mb)	Mapped Reads Number	Mapped Reads %	Average Bases (bp)	Coverage	Average Depth
4 ℃	4.3%	5196.3	35410497	95.01	4969808	100%	1045.6
30 ℃	5.3%	1928.4	13179571	96.92	4969805	100%	388.0

Table S4. Nucleotide sequences of the primers or primer pairs used in this study. f indicates forward primer and r indicates reverse primer. Expected size of PCR products in the wild-types or in the mutants are also shown, respectively.

Primer /Purpose	Primer Sequence (listed 5' to 3')	Expected Size (bp)
**PCR for screening CP4-57So excision**		
SmpB-f	CCTTTATGCATGGCTGTACC	
SO_1439-r	CCTTATCCTTGTCATCGGGT	985(ΔCP4So)
SO_1444-f	GCCCCACAAATAGTCTTCACTGC	
SO_1444-r	CCATATCCGGCTTCTTTTTGGTG	293
IntA-f	CGGGATCCATGGCAAGACTTACTAAGCCG	
IntA-r	CCCAAGCTTTTATTGAGCTACAAGCCTTA	1265
SmpB-r	AACCCGTAGCGTAAAGATAACC	362
Circle-f	AACCATACAGCCACAAGACCG	
Circle-r	GCCAATACAACACCTTAGCACC	830 (CP4So circle)
**qPCR**		
CP4So-f	AGCGCCTTGGATGTAGGTTTT	193
CP4So-r	TCGCTTGGAATACCATTTTGAA	
LambdaSo-f	ACTTTTGCATCGGCTTCGAT	200
LambdaSo-r	AGGCGGTATTTTCGGGTTTG	
MuSo1-f	TCGCAAGGCAATTACGTGAA	220
MuSo1-r	AATTCTGTGACCATCGCCATTA	
MuSo2-f	TGTAAACGGGATCGAAGTGTTG	210
MuSo2-r	TGGCAAGTGGGTAAGCGAAT	
gyrB_f	CGGATGCTTTACCACCCAAT	200
gyrB_r	TGCGTGTCGATACCGTGTTT	
**qRT-PCR**		
alpA	CACAACTCTCACAAGCAATTCCA	188
	GCAGCCACTGTTCGACTTCTT	
intA	GCAGGGCTTTGATCCTGATG	178
	TCTGGCATTATGCGCAAGAC	
hns	TGGAAGCAGAAGAGCTGCAA	175

	TCGCCGTCAACTTCAATTTG	
rrsE	TTCGCGTTGCATCGAATTAA	189
	CCTGGTAGTCCACGCCGTAA	
SO_3012	CGCCAGCTCAACTTGCAAA	150
	CGTTCTCGTAAATCCACGCTTTA	
**Construction of in-frame deletion mutants**		
ssrA-5-O	GGGGACAAGTTTGTACAAAAAAGCAGGCTCTTGGCACTCTCAATCTAAGGAAACA	
ssrA-5-I	GGTCCGGGTTCGCTATCTATCAAGAAAGTTGATTGTAACCCGTAGCG	
ssrA-3-O	GGGACCACTTTGTACAAGAAAGCTGGGTTAATACCTGATAGGGGATTGGCA	
ssrA-3-I	ATAGATAGCGAACCCGGACCAATGATAGAACGAAGACGTCCTAG	
alpA-5-O	GGGGACAAGTTTGTACAAAAAAGCAGGCTTGTAGAGTATCTTAGGTTAAGCAACG	
alpA-5-I	GGTCCGGGTTCGCTATCTATAATCGTCCTCAGAGTTGTGAATAAAC	
alpA-3-O	GGGACCACTTTGTACAAGAAAGCTGGGTGTTAAATTTCTCAATCGCCTTCTT	
alpA-3-I	ATAGATAGCGAACCCGGACCTGCTGATCTTAAAAAGTCACTAAC	
intA-5-O	GGGGACAAGTTTGTACAAAAAAGCAGGCTACCCATGTGGTCTGCGATCCTATA	
intA-5-I	GGTCCGGGTTCGCTATCTATAACAACACCTTAGCACCAATATTT	
intA-3-O	GGGACCACTTTGTACAAGAAAGCTGGGTAGGGGGGTACATCAGGTTTCAGTG	
intA-3-I	ATAGATAGCGAACCCGGACCCGAGAAATGATGACCTGGTGGAGT	
hns-5-O	GGGGACAAGTTTGTACAAAAAAGCAGGCTGCTTTTACCCTGACCTTCAC	
hns-5-I	GGACAATCCGCCTGTTTTCGCTATTTTCGTCCCACTCTAT	
hns-3-O	GGGACCACTTTGTACAAGAAAGCTGGGTCATAATCGGTAAAGGGATCG	
hns-3-I	CGAAAACAGGCGGATTGTCCTCCAGAACTGTGTTGGTAACA	
**Confirmation of mutants by PCR followed by DNA sequencing**		
ssrA-f	CGACAAGCGTGAAGATACCA	
ssrA-r	GCCATAACAACACCTTAGCACC	
hns-f	TAGGGTTTCCTTCCTACA	
hns-r	TGTTGTTTATCAGCATTT	
alpA-f	CTGACATTCCCGAAACAAG	
alpA-r	ATACATTAACTGCCCCCTG	
intA-f	GCCTTGGATGTAGGTTTTC	
intA-r	GTGTCAGGTTCATTGTCGT	

**Constrcution of expression vectors**		
alpA-F	GGAATTCATGAACACAACTCTCACAAGC	229
alpA-R	CCCAAGCTTTCAGCAAAATCTTCTACCTT	
intA-F	CGGGATCCATGGCAAGACTTACTAAGCCG	1265
intA-R	CCCAAGCTTTTATTGAGCTACAAGCCTTA	
ssrA-F	GGAATTCGGGGGCGATTCTGGATTCGACA	371
ssrA-R	CCCAAGCTTTGGTGGAGGCGGCGGGACTTGA	
ssrA-delU-R	CCCAAGCTTTGGTGGGGCGGCGGGACTTGAAC	370
pET28b-hns-F	CTAGCCATGGGCATGAGCGAATTTTTAGAAATATTAACTCACGG	439
pET28b-hns-R	TAAGAATGCGGCCGCTTAGTGGTGGTGGTGGTGGTGGATTAAGAAATCATCCATAGAA	
**Amplify fragments for EMSA**		
alpA promoter-f	GATTTTCTAGTGACAGAAGGTTTTAGCG	237
alpA promoter-r	GTGAGAGTTGTGTTCATAATCGTCCTC	
SO_3012 promoter-f	TAGCGATGAAATGGCTTTTTGGG	172
SO_3012 promoter-r	CTGCTGCACCACTTTACCCTGC	

Fig. S1 Confirmation of CP4So excision by PCR. (a) Schematic of the CP4So excision induced by temperature downshift. Site-specific recombination occurs through the cross-over between *att*L and *att*R sites to generate the ΔCP4So strain and phage-like circle CP4So^{circle} (which is lost subsequently). Location of primer pairs SmpB-f/SO_1439-r, and circle-f/circle-r are also shown. **(b) Deletion of CP4So was confirmed by PCR**. Lanes 1, 3, 5 and 7 used DNAs from the wild-type strain, and lanes 2, 4, 6 and 8 used DNAs from the ΔCP4So strain. Primer pair SmpB-f/SO_1439-r was used to amplify a 985 bp fragment only when the whole CP4So was removed (marked with an arrow). Primer pairs (SO_1444-f/-r and IntA-f/-r) were used to detect the presence of CP4So-specific genes (PCR products are 293 bp and 1265 bp in the wild-type strain, respectively). As control, primer pair SmpB-f/-r was used to amplify a 362 bp fragment in *smpB* gene outside CP4So. (**c**) **Circularized CP4So prophage was confirmed by PCR**. Lanes 1 and 2 used DNAs from the wild-type cells induced by AlpA and non-induced AlpA, respectively. Primer pair Circle-f/-r was used to amplify an 830 bp fragment only when the excised CP4So was circularized (marked with an arrow).

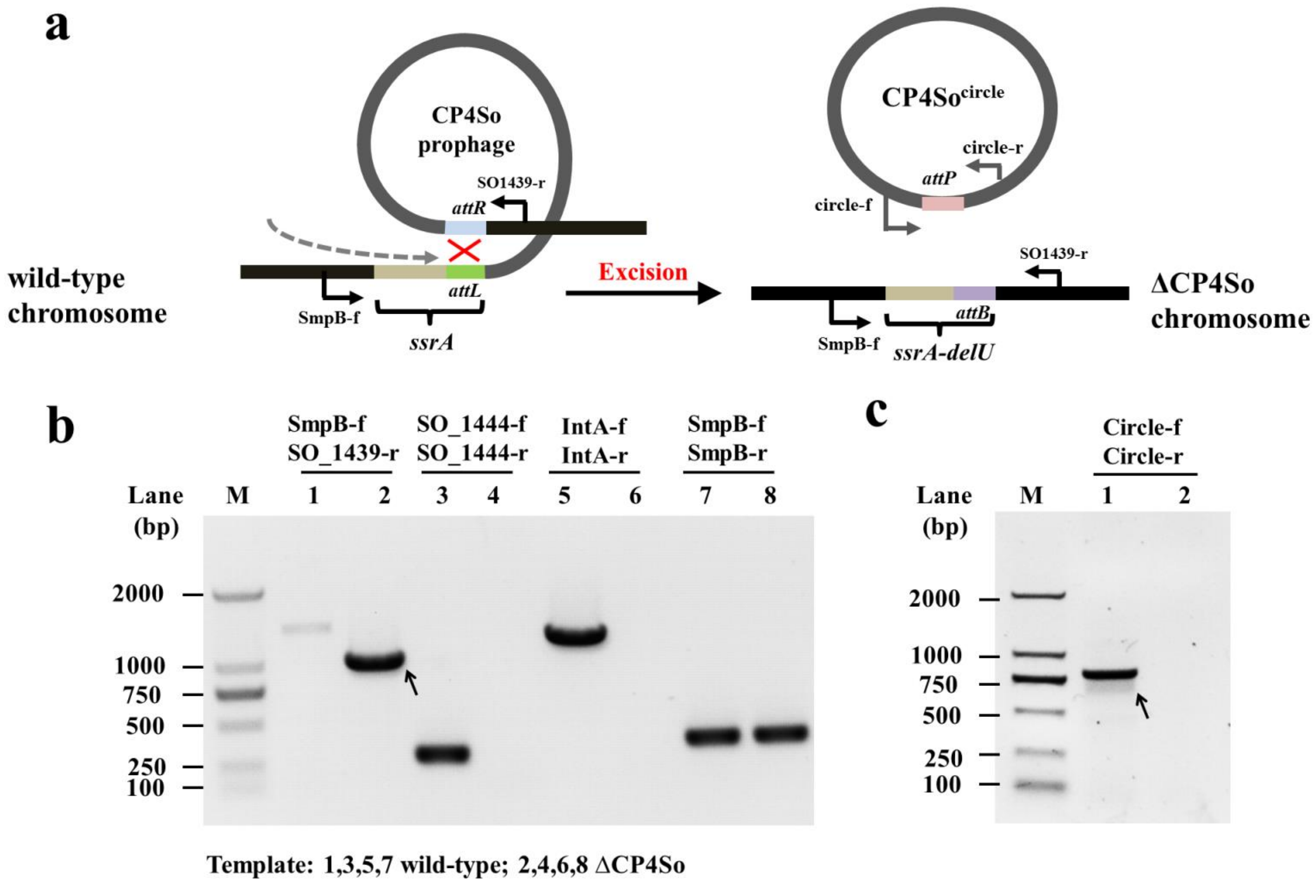

Fig. S2 Excision rates of CP4So when exposed to low temperatures. Excision rates of CP4So in *S. oneidensis* at different time points after exposed to the cold temperatures from exponentially growing cells at 30 ℃ of OD 600 nm of around 1.0. Data are from three independent experiments and one standard deviation is shown.

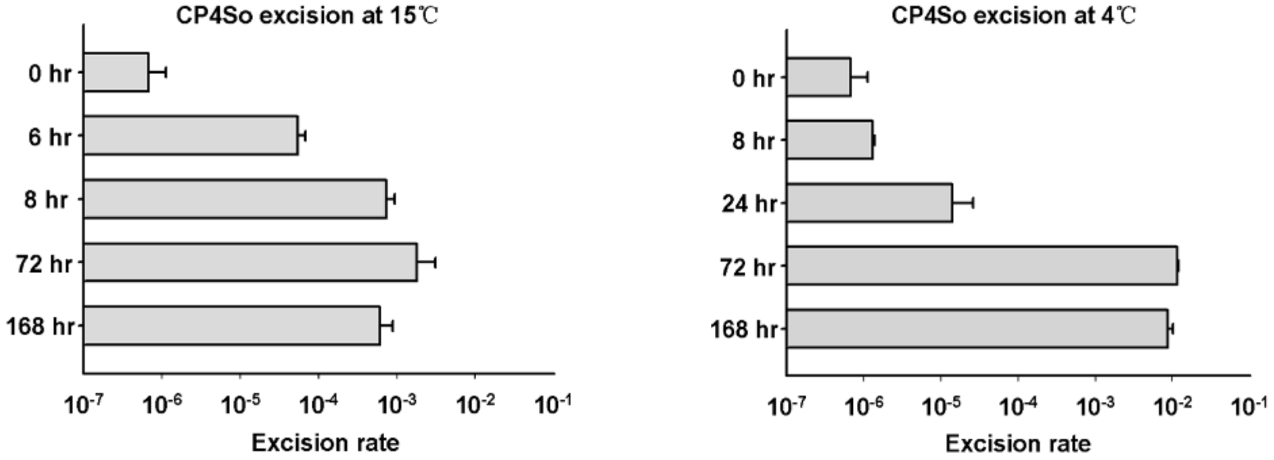

Fig. S3 Excision rates of CP4So and LambdaSo in *S. oneidensis* upon initiation of the SOS response. The excision rate of was measured upon initiation of the SOS response with the addition of 10 μg/mL mitomycin C (MMC). Data are from three independent experiments and one standard deviation is shown.

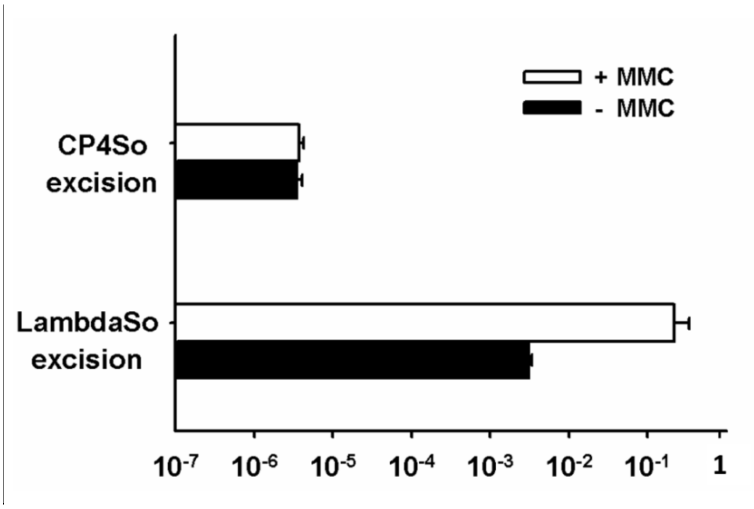

Fig. S4 SsrA does not seem to function at cold temperatures. Survival of cells when exposed to sublethal concentrations of gentamicin (1 μg/mL) for the wild-type, Δ*ssrA* and ΔCP4So strains at different temperatures. Data are from three independent cultures and only representative images are shown here.

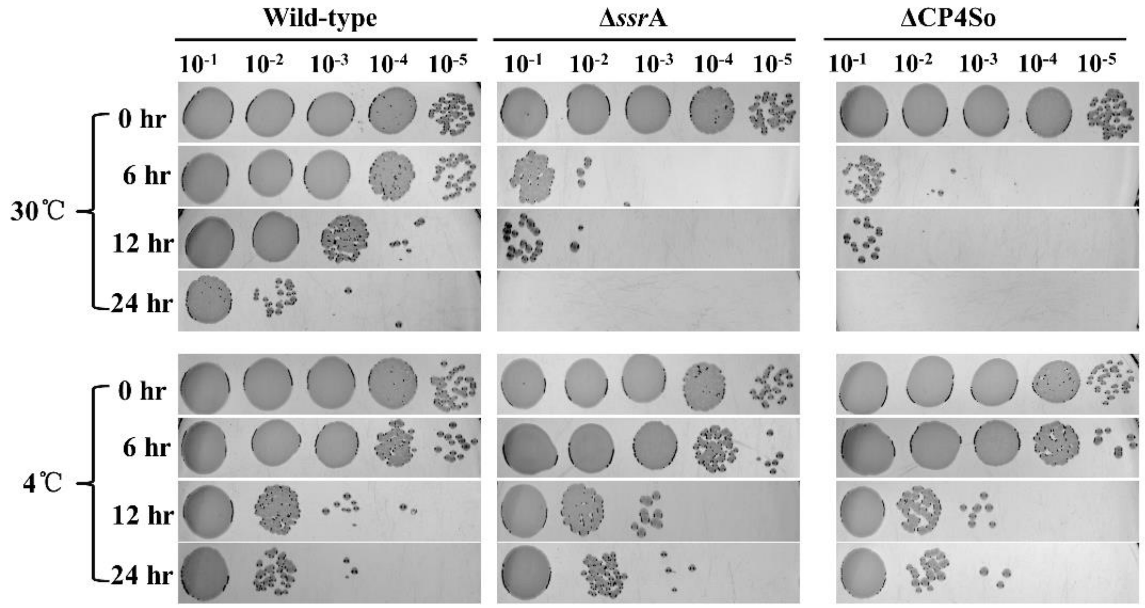

Fig. S5 Excision of CP4So enhances survival at cold temperatures. (a) Viability of the wild-type, Δ*ssrA* and ΔCP4So cells cultured at 4 ℃ for 14 days was detected by LIVE/DEAD staining method. **(b)** Viability of the wild-type, Δ*ssrA* and ΔCP4So strains was measured by a turbidity at 600 nm after transferring the cells grown at 4 ℃ to 30 ℃ with fresh LB medium. Data are from three independent cultures. One standard deviation is shown in **b**, and representative images were shown in **a**.

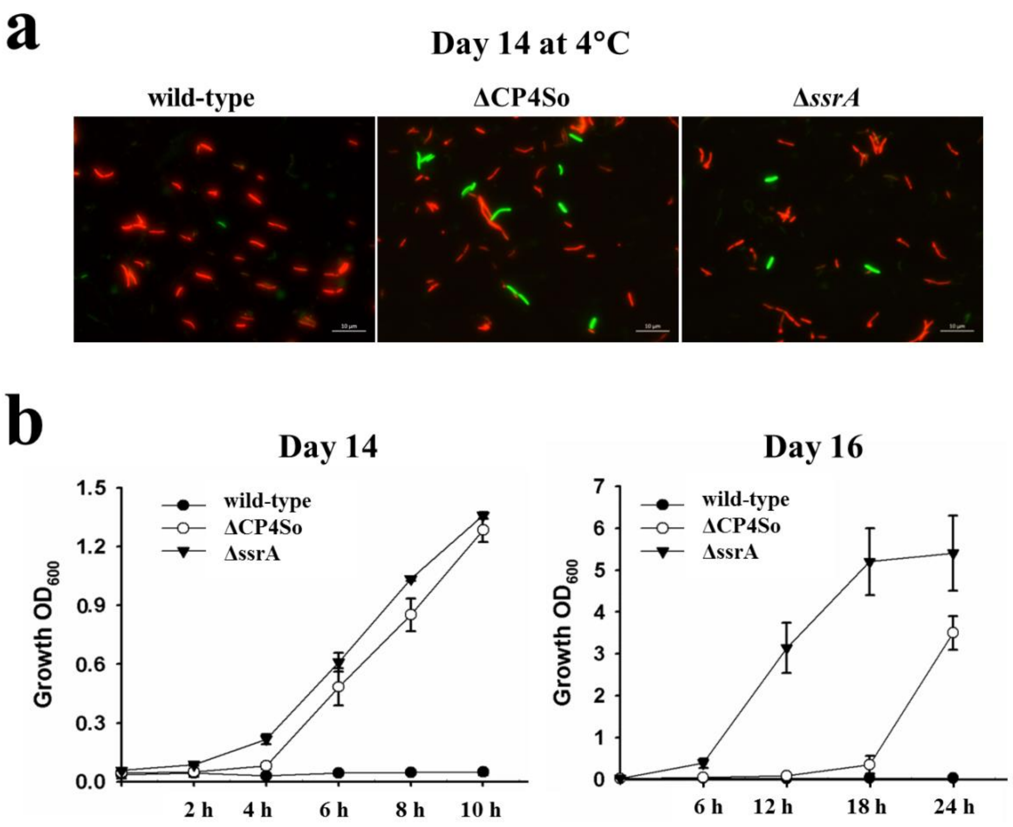

Fig. S6 Comparison of amino acid sequence of the putative excisionase AlpA in the prophage CP4So of *S. oneidensis* MR-1 and prophage CP4-57 of *E. coli* K-12 BW25113.

```
                    *        20         *        40         *        60         *
MR-1    : MNTTLTSNSIIRLKTLVQLTGLSRSTIYDKLNPKSPRFDPLFPRKVQLGARAVGWYMQEVEQWLQGR-RFC : 70
BW25113 : MNN-RSAVRILRLPAVIQKTGMARATIYDWLNPKSPRYDATFPKKRMLGVKSVGWIEAEIDEWLSQRCKLI : 70
          MN        I RL    Q TG   R TIYD LNPKSPR D  FP K  LG   VGW   E   WL  R
```

Fig. S7 Promoter sequences and the open reading frame sequences of *alpA* gene (a) and *SO_3012* gene (b). (**a**) Primer pair AlpA-promoter-f/r used to amplify the fragments containing promoter of *alpA* was underlined and highlighted with blue. The two putative H-NS binding sites (GATAATG) were labeled with yellow. (**b**) Primer pair SO_3012 promoter-f/r used to amplify the fragments containing promoter of *SO_3012* was underlined and highlighted with blue. The numbers denote the nucleotide positions from the translation start site. The start and stop codon of each gene were labeled in green and red, respectively.

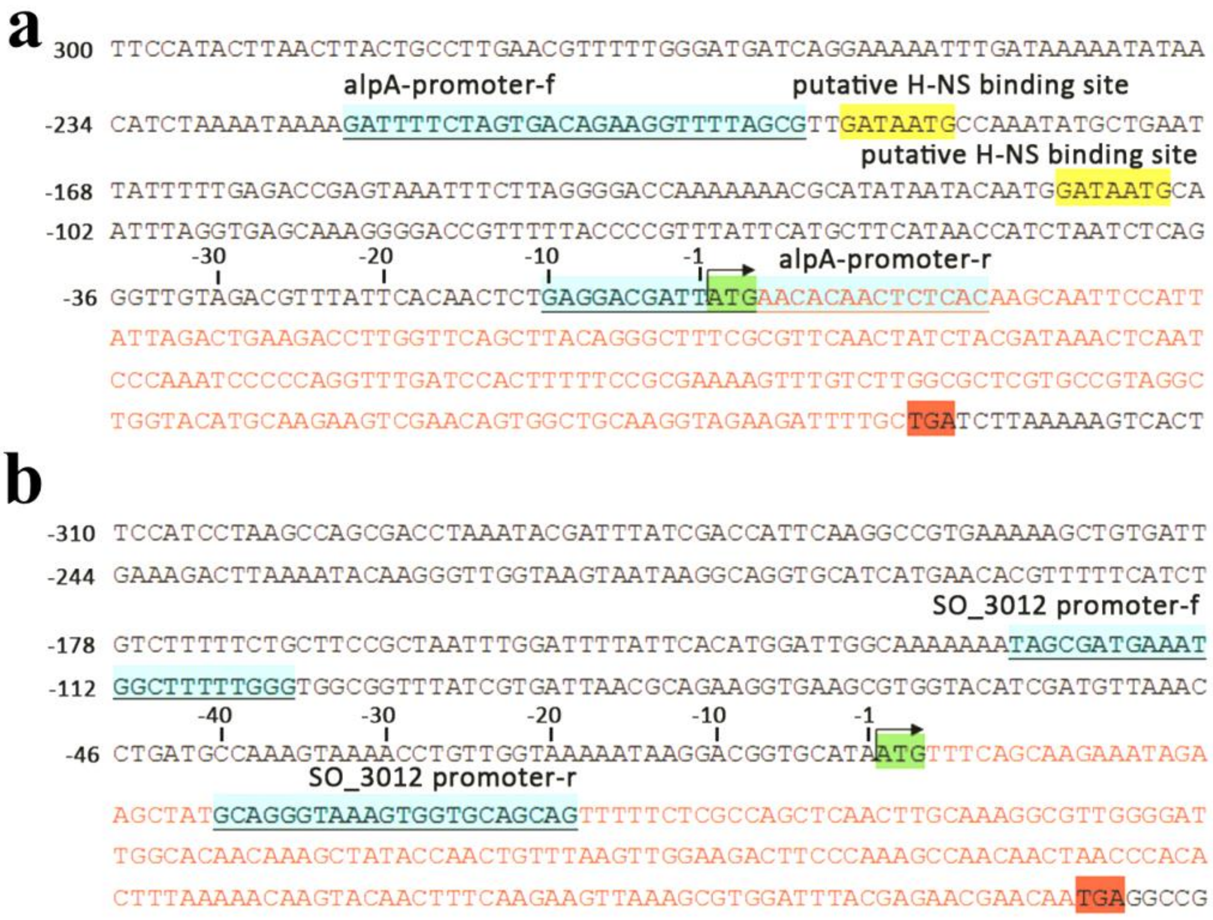

Fig. S8 EMSA of H-NS and the promoter of excisionase gene *alpA*. The affinity of H-NS binding to the promoter of *alpA* is comparable at different temperatures.

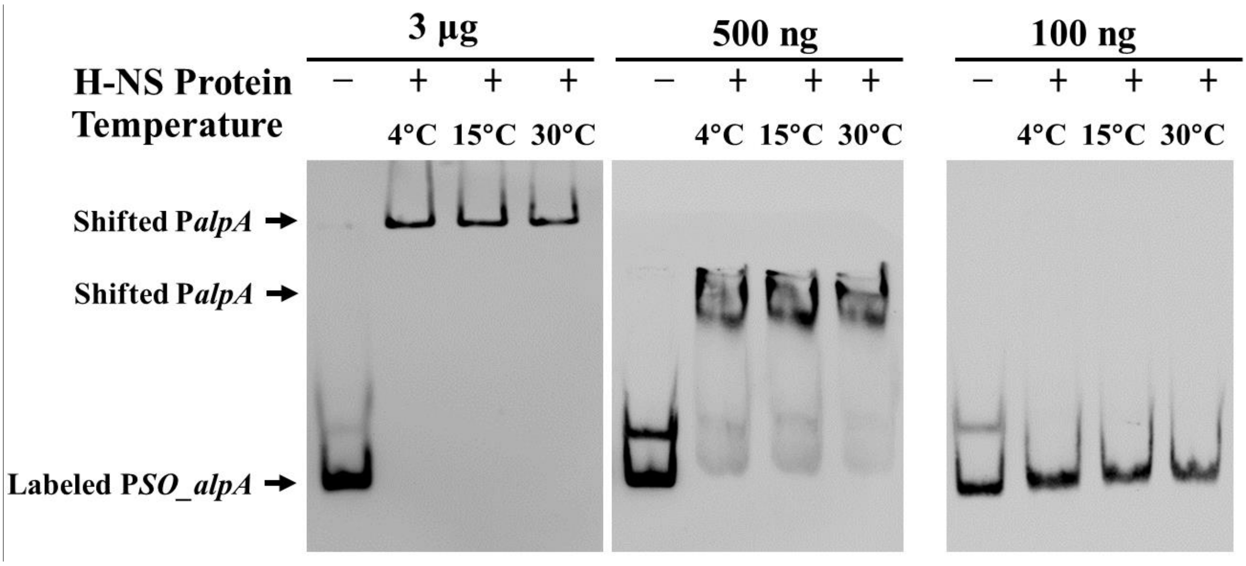

Fig. S9 Mobile elements inserted in the tmRNA gene in different *Shewanlla* strains.

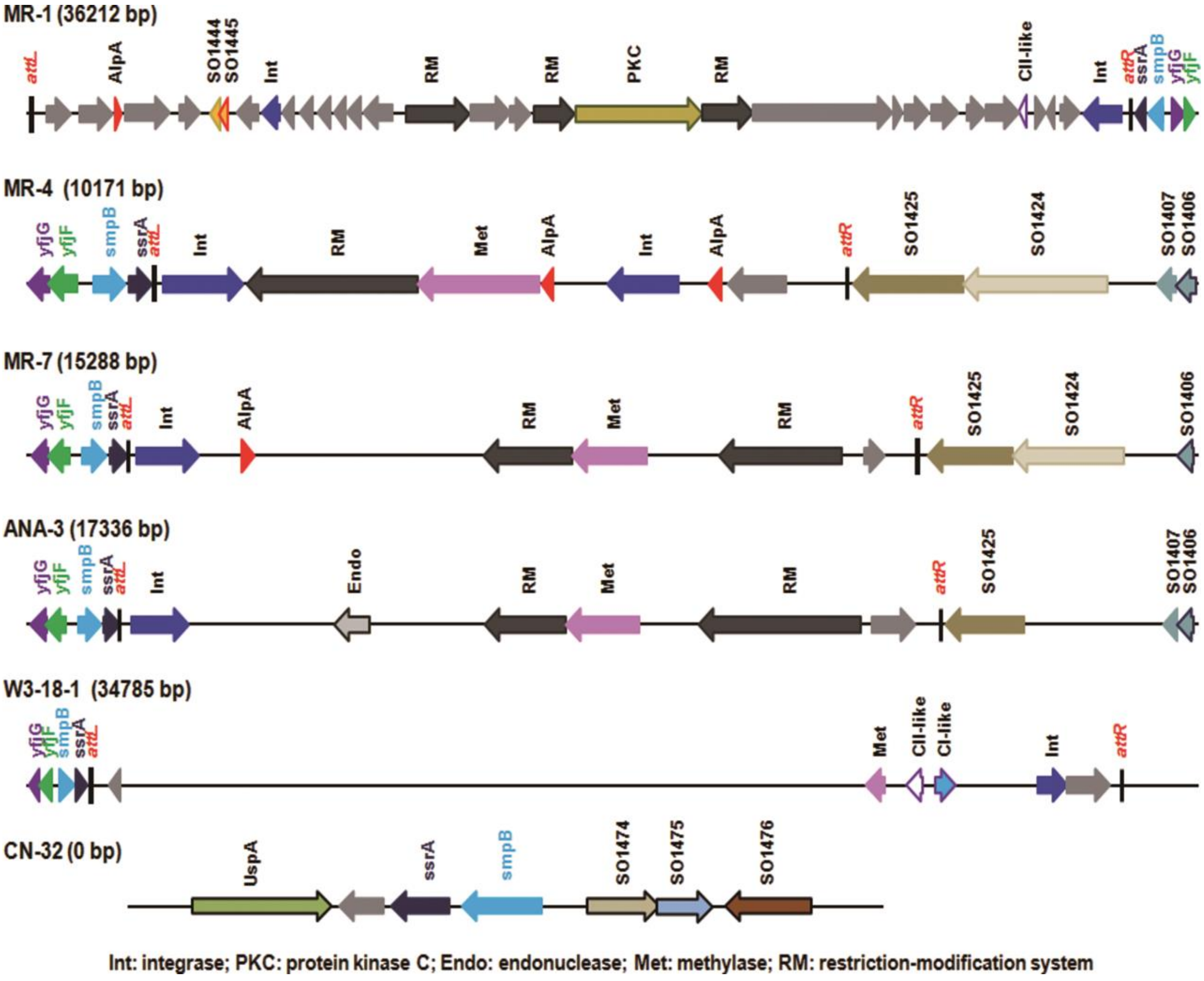

Fig. S10 Standard curve for calibration of primer pair (CP4So-f and CP4So-r) used for measuring excision rate of CP4So in *S. oneidensis* via qPCR. Mixed population of wild-type and ΔCP4 DNAs were used as template to generate a standard curve. Data are from two independent cultures.

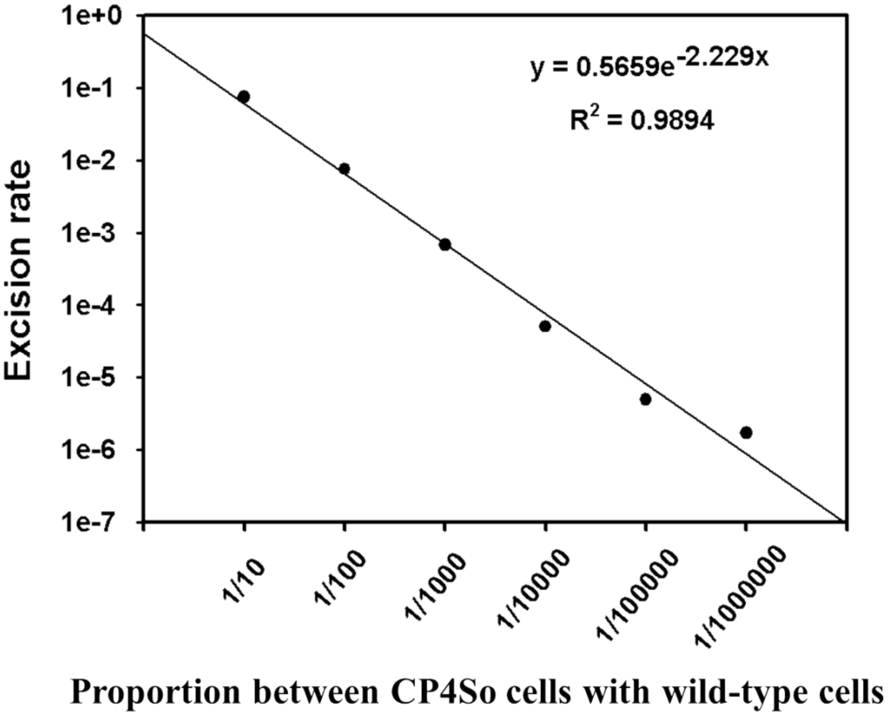

Supplement: Supplementary Information [file ismej201685x1.pdf]
